# Exploring the Metabolic and Endocrine Preconditioning Associated with Thyroid Disorders: Risk Assessment and Association with Acne Severity

**DOI:** 10.3390/ijms25020721

**Published:** 2024-01-05

**Authors:** Alexa Florina Bungau, Delia Mirela Tit, Simona Gabriela Bungau, Cosmin Mihai Vesa, Andrei-Flavius Radu, Ruxandra Cristina Marin, Laura Maria Endres, Lavinia-Cristina Moleriu

**Affiliations:** 1 Doctoral School of Biomedical Sciences, Faculty of Medicine and Pharmacy, University of Oradea, 410087 Oradea, Romania; pradaalexaflorina@gmail.com (A.F.B.); dtit@uoradea.ro (D.M.T.); andreiflavius.radu@uoradea.ro (A.-F.R.); 2 Department of Preclinical Disciplines, Faculty of Medicine and Pharmacy, University of Oradea, 410073 Oradea, Romania; 3 Department of Pharmacy, Faculty of Medicine and Pharmacy, University of Oradea, 410028 Oradea, Romania; 4 Fundeni Clinical Hospital, 022328 Bucharest, Romania; rcfhm@yahoo.com; 5 Department of Psycho-Neurosciences and Recovery, Faculty of Medicine and Pharmacy, University of Oradea, 410073 Oradea, Romania; lendres@uoradea.ro; 6 Department III of Functional Sciences, “Victor Babes” University of Medicine and Pharmacy, 300041 Timisoara, Romania; moleriu.lavinia@umft.ro

**Keywords:** acne, metabolic preconditioning, endocrine preconditioning, hormonal imbalance, metabolic syndrome

## Abstract

Metabolic preconditioning, characterized by conditions like obesity and insulin resistance syndrome, disrupts hormonal balance. Elevated androgen levels stimulate excessive sebum production and follicular cell proliferation, leading to acne lesions. Similarly, thyroid hormone imbalances affect sebaceous gland activity, epidermal lipid composition, and skin cell turnover, impacting acne occurrence and severity. This study aimed to assess the potential contribution of metabolic and endocrine preconditions to acne development. A total of 389 patients diagnosed with acne were included and divided into three groups: the metabolic precondition group (MPG, N = 163, 41.9%), the endocrine precondition group (EPG, N = 162, 41.65%), and the control group (CG, N = 89, 22.88%). Data related to the degree of acne severity and comorbidities of interest were collected from the patients’ medical records. In the groups with concomitant diseases, moderate and severe acne were significantly more prevalent (56.44% and 41.10% in MPG, and 35.80% and 61.11% in EPG) compared to the control group (5.61% and 4.89%). The most prevalent preconditions observed were insulin resistance syndrome in MPG (63.8%) and autoimmune thyroiditis in EPG (95.06%). Significant age-related differences in acne severity were found across all study groups (*p* < 0.05). In MPG, the age variable was significantly higher in the presence of mild acne, while in EPG, the age variable was significantly lower for the mild acne group. A positive association was observed between the severity of acne and insulin resistance syndrome, obesity, autoimmune thyroiditis, and hypothyroidism (*p* < 0.05). Risk analysis indicated a significantly higher risk (RR > 1, 95% CI RR > 1, *p* < 0.001) of developing moderate and severe acne in the presence of these preconditions. The presence of both metabolic and endocrine preconditions significantly increased the likelihood of developing severe acne, leading to the hypothesis that both conditions may be contributing factors to the development of acne.

## 1. Introduction

Over 85% of youngsters suffer from acne, one of the most common skin problems [1,2]. Although some people continue to experience acne into their 40 s and 50 s, it normally begins during puberty and eventually subsides by the time the person is 20 [3]. Besides mainly affecting adolescents, acne represents a chronic, inflammatory condition of the pilosebaceous region that can also continue to develop in adults. Genetic factors, stress, androgens, excess sweating, and various pathologic preconditions all play a role in the onset and/or severity of acne [4]. In addition to endocrine conditions like Cushing’s syndrome and polycystic ovarian syndrome, it is also known that corticosteroids, oral contraceptives, iodides, bromides, lithium, and chemicals like dioxins can cause acne outbreaks [5]. 

The primary etiopathogenic factors for acne, which is a multifactorial disorder, are follicular hyperkeratinization, increased sebum production, higher bacterial (*Propionibacterium acnes*) development in the pilosebaceous unit, and inflammation, although the exact process is still unclear. These factors combined result in the release of a number of proinflammatory mediators, including IL-1, IL-8, tumor necrosis factor (TNF)- α, and IL-10 [6].

Acne’s clinical symptoms are a collection of symptoms connected to enlarged, inflammatory, or scarred pilosebaceous units. The symptom that manifests most frequently is seborrhea. Usually, the face, neck, upper back, and chest are the sites of lesions [1]. 

Pustules, papules, nodules, and cysts are the many types of inflammatory lesions that can appear on distended pilosebaceous units, which can appear as open or closed comedones. The distribution (back, chest, upper arms), kind, and quantity of lesions (comedones, papules, pustules, and nodules), as well as the presence or absence of scarring, must all be taken into account when determining the severity of acne [7]. Macular pigmentation and scars (hypertrophic, keloids, ice pick scars, depressed fibrotic and atrophic macules, and perifollicular elastolysis) are examples of post-inflammatory lesions that can also develop. In skin with pigment, post-inflammatory hyperpigmentation is frequently observed [8].

In recent years, debates have been observed over the connection between nutrition and acne [9]. Studies that have been conducted throughout this period to support this idea, however, have produced conflicting findings [10]. Disturbances in systemic metabolism frequently led to cutaneous symptoms, and vice versa. A variety of hormonal abnormalities are brought on by the accumulation of extra adipose tissue and insulin resistance in metabolic syndrome (MetS) [11]. 

The term “metabolic syndrome” refers to a group of risk factors that have metabolic origins and are associated with a higher risk of developing type 2 diabetes mellitus and cardiovascular disease. Atherogenic dyslipidemia, central obesity, high blood pressure, and higher plasma glucose levels are some of these risk factors [12]. Metabolic syndrome guidelines, provided by the International Diabetes Federation (IDF) in 2005, considered central obesity (specified as waist circumference but presumed if BMI > 30 kg/m^2^) with values particular to each ethnic group, plus two of the following criteria: triglyceride levels of 150 mg/dL or higher; HDL cholesterol levels (in men < 40 mg/dL and women < 50 mg/dL, respectively); blood pressure of 130/85 mmHg or higher; fasting glucose levels of 100 mg/dL or above [13]. In the pathogenesis of MetS and acne, inflammatory markers like TNF-α, IL-17, IL-23, and oxidative stress have shown a possible correlation [14]. 

Being a chronic inflammatory skin condition, acne may also have a substantial correlation with insulin resistance (IR) [15]. IR examination seems crucial for both detecting this disease and developing a therapy plan. The same signal transduction pathways—mammalian target of rapamycin kinase 1 (mTORC1) and insulin-like growth factor-1 (IGF-1)—are involved in both diseases [16].

The elevated incidence rates of teenage acne cannot be attributed to genetic predisposition but rather to the impact of a Western diet that overstimulates the growth factor- and nutrient-sensitive kinase mTORC1, a major metabolic conductor. In comparison with subjects who did not have acne, there was a rise in mTORC1 activity in the sebaceous glands and lesion skin of acne patients. Neurodegenerative disorders, type 2 diabetes mellitus, insulin resistance, cancer, and obesity are all known to relate to elevated mTORC1 signaling. The sebaceous follicle MetS is represented by acne vulgaris, one of the mTORC1-driven illnesses of civilization [16].

In clinical settings, thyroid abnormalities are very common; they are linked to a variety of illnesses, some of which may or may not have the same etiology. The skin is one of the organs that best displays this wide variety of clinical symptoms [17]. A variety of intricate mechanisms enable the endocrine and integumentary systems to interact. It is unclear how thyroid hormones affect the sebaceous glands. Autoimmune thyroid disorder susceptibility is influenced by a complex interplay of environmental and genetic variables. Acne vulgaris and chronic idiopathic urticaria are two chronic inflammatory skin diseases in which thyroid autoimmunity has been found [18]. IL-1ß may be crucial in the emergence of inflammation in acne as well as in autoimmune thyroid illnesses. In human sebocytes, it has been demonstrated that IL-1ß feeds the inflammation that *C. acnes* started [19].

The main purpose of this investigation is to evaluate the potential contribution of metabolic and endocrine thyroid-related preconditions to the development of acne. Additionally, a risk analysis was conducted to determine the likelihood that individuals with metabolic and endocrine disorders may experience severe types of acne. Moreover, it is the first study that, as far as we are aware, assesses the effect of metabolic and endocrine preconditioning on the severity of acne.

## 2. Results

Table 1 lists the demographic and clinical characteristics of the participants in the current investigation. Female patients outnumbered male patients by a ratio of 5.71:1 and urban patients outnumbered rural patients by a ratio of 3.69:1. The highest proportion of male patients (26.38%) was registered in the MPG, followed by the CG (7.87), while in the EPG only 4.32% of patients were men. Over 75% of the patients who were enrolled were older than 25 years. The lowest proportion of participants over this age (57.7%) was observed in the group with metabolic preconditioning, suggesting a higher incidence of this pathology in younger patients. Nearly 97% of the patients with endocrine problems were older than 25, compared to 78.7% of the subjects in the control group.

In the groups with concomitant diseases, moderate and severe acne were substantially more prevalent (56.44% and 41.10%, respectively, in the MPG and 35.80% and 61.11%, respectively, in the EPG), than in the control group (5.61% and 4.89%, respectively). Insulin resistance syndrome was the most prevalent metabolic precondition (63.80%), followed by obesity (50.92%). Autoimmune thyroiditis with hypothyroidism was the most prevalent thyroid precondition (41.98%). Forty-two individuals (14.00% of the total patients with disease) were enrolled in both groups and completed both metabolic and endocrine preconditioning.

By applying a chi square test, insignificant differences were obtained $\left(p>0.05\right)$ for the gender classification regarding the acne severity in the group with sick patients.

**Figure 1:**
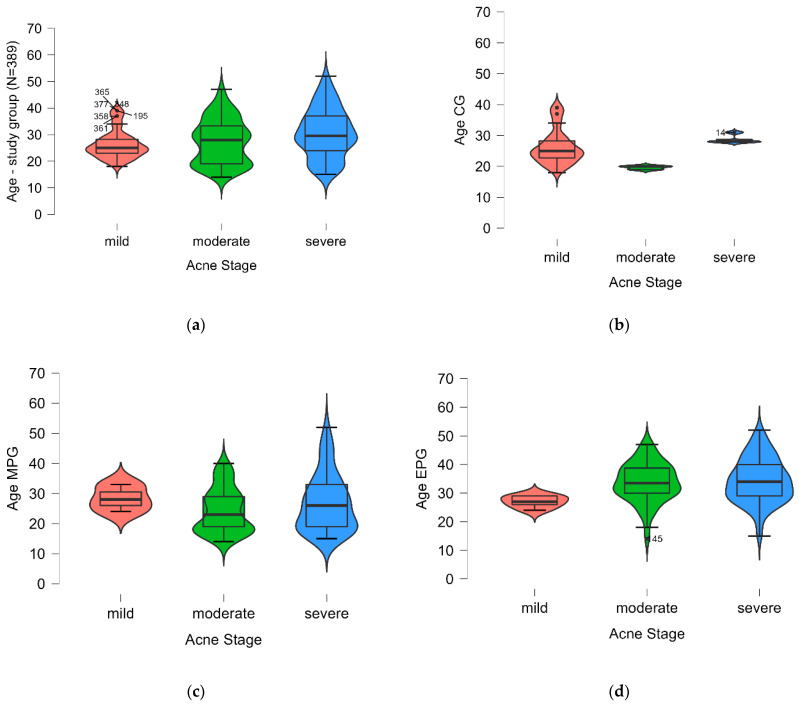
The acne stage distribution presented using a boxplot: (**a**) study group; (**b**) CG; (**c**) MPG; (**d**) EPG. MPG—metabolic preconditioning group, EPG—endocrine preconditioning group, CG—control group.

Observing that the mean age of most patients in the study, from all three groups, was over 25 years old, the study tested if the patients’ age could be associated or not with the stages/severity of acne and the presence of MP and EP. All the results are presented in Table 2 and Figure 1. The mean age was highest in the EPG with severe acne (34.36, 7.74 SD years), whereas it was lowest in the CG with mild acne.

Using the data collected from the descriptive statistics, the Mann–Whitney test was performed to determine whether there were any significant differences in the age of the patients with vulgar acne based on the existence of metabolic preconditioning or endocrine preconditioning. The research showed that participants with metabolic preconditioning were younger than those without metabolic preconditioning, whereas endocrine preconditioning-related thyroid disorders revealed a reverse trend. The results also showed statistically significant differences (*p* < 0.05) in the age of patients with metabolic or endocrine preconditioning. Testing each disease individually, the study obtained significant differences (*p* < 0.05) for insulin resistance syndrome, HBP, dyslipidemia, and obesity in the MPG, and for autoimmune thyroiditis, autoimmune thyroiditis with hypothyroidism, and autoimmune thyroiditis with hyperthyroidism in the EPG (Table 3).

**Figure 2:**
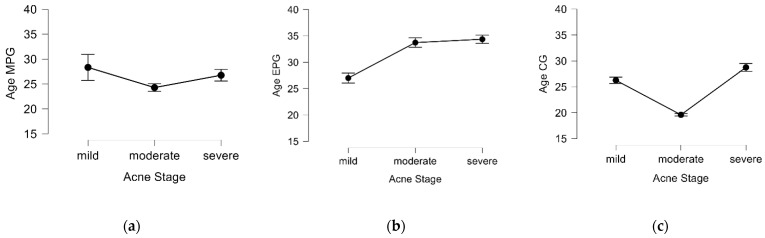
The graphical representation of the Kruskal—Wallis test showing the dependence of age and acne stages in all three groups: (**a**) MPG (Statistics = 2.251, *p* = 0.048); (**b**) EPG (Statistics = 6.595, *p* = 0.037); (**c**) CG (Statistics = 13.867, *p* < 0.001). MPG, metabolic preconditioning group; EPG, endocrine preconditioning group; CG, control group.

A Kruskal–Wallis test was used to further examine the data in order to see whether there was an association between the age of the patients and the severity of the acne in each of the three groups. There were statistically significant age-related differences in acne severity across all study groups $\left(p<0.05\right)$. In the MPG, the age variable was significantly higher in the presence of mild acne, while in the EPG, the age variable was significantly lower for the mild acne group. In the CG, the age was significantly higher in the severe acne sample of patients. The statistical results are shown in Figure 2.

In the two study groups (MPG and EPG), most of the participants had moderate to severe acne. Moderate acne affected 56.44% of patients in the MPG. Of these, 68.49% had insulin resistance syndrome, and 71.74% were obese. The fact that this group exhibited a prevalence of severe acne (41.1%), insulin resistance (61.19%), and obesity (25.37%) shows a strong association between these disorders and the severity of acne. In the EPG, 35.8% of patients suffered from moderate acne and 61.11% from severe acne, of which 44.83% and 51.52%, respectively, had autoimmune thyroiditis, and 41.38% and 44.44%, respectively, were diagnosed with autoimmune thyroiditis and hypothyroidism (Table 4).

Using a chi square test, the statistical association between the acne stage and the presence/absence of metabolic and endocrine preconditions was tested. The results showed a positive association, meaning that if these preconditions were present, the stage of acne was significantly more severe $\left(p<0.05\right)$, regardless of age. Extending the statistical analysis to each pathology from the two groups, the study obtained statistically significant results (*p* < 0.05), and a positive association between glucose, insulin resistance syndrome, obesity, autoimmune thyroiditis, and autoimmune thyroiditis with hypothyroidism and the severity of acne. Applying the same analysis to the age subgroups (12 to 25 years—adolescent acne, and >25 years—adult acne), a positive association was found between insulin resistance syndrome and obesity and the severity of acne in both age subgroups (12–25 years and over 25 years old). In the EPG, statistically significant results were found only in the subgroup of patients over 25 years old. Patients diagnosed with autoimmune thyroiditis and autoimmune thyroiditis with hypothyroidism are at a higher risk of developing a more severe stage of acne, with a positive association being found between those parameters (Table 5). 

**Figure 3:**
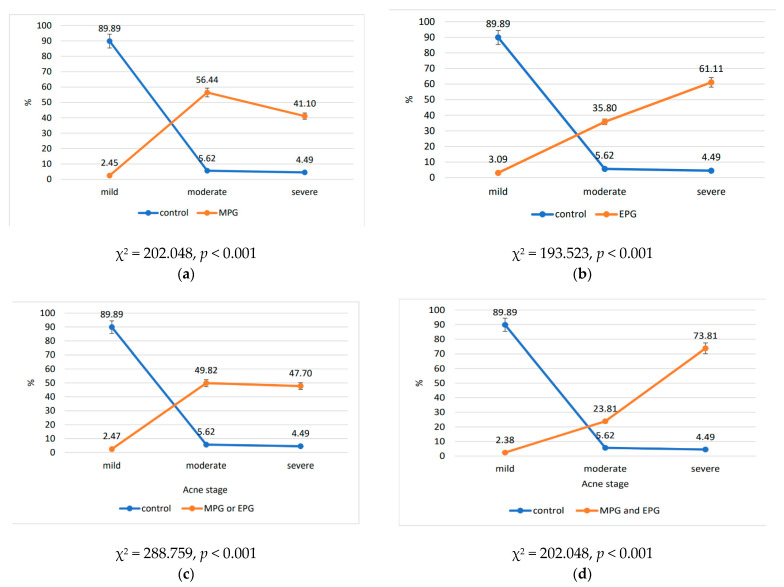
The associations between the acne stages and metabolic and endocrine diseases, comparing each studied group vs. the control group: (**a**) MPG; (**b**) EPG; (**c**) MPG or EPG; (**d**) MPG and EPG. MPG, metabolic preconditioning group; EPG, endocrine preconditioning group; CG, control group; MPG or EPG, subgroup with either metabolic or endocrine preconditioning; MPG and EPG, subgroup with both metabolic and endocrine preconditioning.

The ordinal and nominal variables were subjected to a chi square test to assess group differences. In this case, two additional subgroups were created to further the analysis: one with either metabolic or endocrine preconditioning (N = 258, denoted as MPG or EPG), and the other with both forms of preconditioning (N = 42, denoted as MPG and EPG). The outcomes revealed statistically significant differences $\left(p<0.001\right)$. When metabolic and/or endocrine disorders are present, acne severity considerably worsens (Figure 3).

To measure the risk of developing moderate and severe vulgar acne in the presence of metabolic or/and endocrine diseases, a risk analysis was performed using 2×2 tables. Patients with severe or moderate acne were considered diseased and the presence of metabolic or/and endocrine preconditioning was considered a risk factor. The Risk Ratio (RR) parameter was calculated, and the 95% confidence interval (95% CI) was estimated. In all six hypotheses, it was found that there was an extremely high-risk factor $\left(RR>1,\;95\%\;CI\;RR>1,\;p<0.001\right)$ for developing moderate and severe acne in the presence of the preconditions. When patients had both classes of disease, the chance of developing severe acne was much higher (Table 6). 

**Figure 4:**
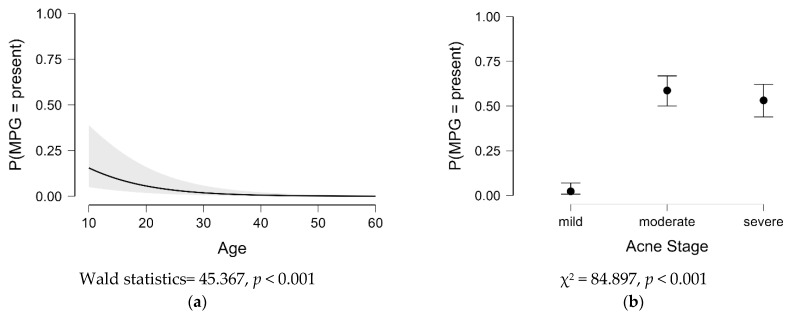

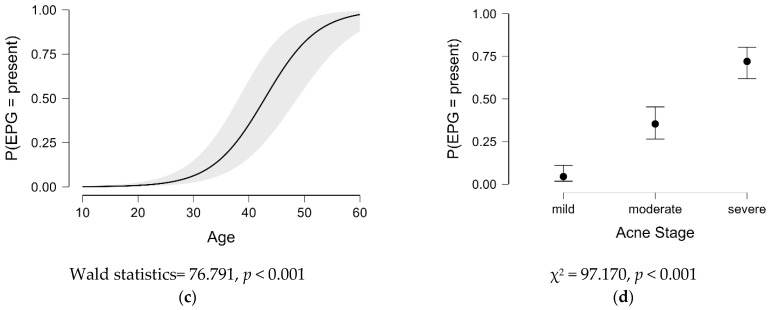
The logistic regression model, for the statistically significant results, by applying the Wald test: (**a**) association between metabolic preconditioning and age; (**b**) association between metabolic preconditioning and acne stages; (**c**) association between endocrine preconditioning and age; (**d**) association between endocrine preconditioning and acne stages. MPG, metabolic preconditioning group; EPG, endocrine preconditioning group; the gray area represents the 95% confidence interval.

Seeing the associations between metabolic and endocrine preconditioning, the acne stages, and the patient’s age, we evaluated the strength of these associations. For ordinal and nominal variables, a logistic regression model was applied, and the outcomes showed a statistically significant association between them (*p* < 0.05). When metabolic preconditioning was present, the risk of developing moderate acne increased significantly, as did the risk of severe acne, mainly in patients under 30 years old. For endocrine preconditioning, the severity of acne increased significantly for moderate acne and severe acne, mainly for patients over 30 years old. The results are plotted in Figure 4.

## 3. Discussion

With an estimated global incidence (for all ages) of 9.38%, acne vulgaris is one of the top ten most prevalent common skin illnesses [20,21]. The prevalence of acne varies across nations and age groups, with estimates ranging from 35% to nearly 100% of adolescents having acne at some point. But the disease can often be found in adults and is caused by various factors [22]. Acne > 25 years old is called post-adolescent or adult acne. Numerous endogenous and exogenous variables play a role in the etiology of adult acne. Hormones produced by the adrenal gland, ovaries, and pancreas, along with inflammatory mechanisms of varying sources and stress, can contribute to sebum oxidation, which may have implications for the onset of acne [23]. Endocrine abnormalities, persistent induction of innate immunity, and genetic predispositions [24] are examples of the former, while cosmetics [25], stress, and tobacco are examples of the latter [26]. In many studies, acne has been noted in two classes: persistent acne and late-onset acne. Persistent acne has been reported as the most common type of adult acne, responsible for 73.2–82% of cases [3]. Unlike adolescent acne, adult acne typically presents as inflammatory papulopustular lesions that develop gradually and generally have a mild–moderate course. According to earlier studies, 61–85% of adult females with acne have mild to moderate cases of the condition [26].

In the present retrospective analysis of 389 patients with acne, of whom over 75% were women over 25 years old, individuals with metabolic and endocrine preconditioning experienced moderate and severe acne substantially more frequently than patients without these comorbidities. Overall, the study’s findings suggested that metabolic and endocrine preconditions related to thyroid disorders might be factors in the development of severe acne. Most studies have found that people who suffer from metabolic or thyroid disorders have an increased prevalence of acne [20]. Obese and overweight people tend to have greater levels of androgen and glycemic loading, which may enhance sebum secretion and encourage the development of acne lesions [27]. El-Akawi et al. found that acne patients in Jordan had noticeably reduced HDL (*p* < 0.001). In addition, TG and LDL levels were considerably higher in people with severe acne when compared to healthy subjects (*p* = 0.004 and *p* < 0.001, respectively) [28]. A high incidence of acne has also been linked to autoimmune thyroiditis, particularly in older women [29]. Epidemiological studies show that women are more likely than men to report experiencing acne beyond the adolescent years [30]. 

This study’s outcomes show that moderate and severe acne were more common in the associated disease groups (56.44 and 41.10%, respectively, in the MPG and 35.80% and 61.11%, respectively, in the EPG) than in the control group (5.61 and 4.89%, respectively). Obesity (50.92%) and insulin resistance syndrome (63.80%) were the most prevalent metabolic preconditions. Forty-two patients (10.79% of the total) from both groups underwent metabolic and endocrine preconditioning. Autoimmune thyroiditis with hypothyroidism was the most prevalent thyroid precondition (41.98%) in the EPG.

Findings from previous studies showed that the greater prevalence of acne comorbidity may be caused by factors such as common dietary structure [9], genetic predisposition, immune–inflammatory pathways, and the influence of excessive testosterone levels [31]. For instance, people with acne are more likely to experience symptoms like anxiety and insomnia, which in turn influence obesity and cardiovascular disease. The same gene locus, RETN-420, regulates metabolic diseases such as acne, obesity, diabetes, and others [32]. It is generally recognized that a kinase pathway known as the target of mammalian mTORC1 can lead to the development of insulin resistance, obesity, and type 2 diabetes [33,34], which is also one of the important mechanisms of acne pathogenesis. Comorbidities, such as obesity, hyperlipidemia, hypertension, and diabetes, which are all high-risk factors for cardiovascular disease, can interact with one another [35]. However, when considering each disease present, this study obtained significant differences (*p* < 0.05) between the patients’ age and the prevalence of insulin resistance syndrome, HBP, dyslipidemia, obesity, autoimmune thyroiditis, autoimmune thyroiditis with hypothyroidism, and autoimmune thyroiditis with hyperthyroidism as metabolic and endocrine preconditions of acne. 

Over 50% of patients under the age of 25 (59.14%) experienced insulin resistance syndrome, which was followed by obesity (40.86%), showing a higher incidence of these diseases in younger patients. Overall, 4.3% of the patients under 25 years old experienced autoimmune thyroiditis, while hypothyroidism was diagnosed in 3.22% of the patients. A Kruskal–Wallis test applied in this research showed that in every studied group (CP, MPG, and EPG), there were statistically significant age-related differences in acne severity (*p* < 0.05). In the group with endocrine preconditioning-related thyroid disorders, patients with severe acne tended to be older, whereas those with metabolic preconditioning were frequently younger. Obesity, at any age, can lower the plasma level of the sex hormone-binding globulin (SHBG), which raises the level of free testosterone and the inflammatory cytokines that cause acne. Other extra triggers include insulin resistance and hyperinsulinemia [36]. Therefore, additional research is required to determine this relationship, especially divided by age category. 

Furthermore, by applying the chi square test to examine the relationship between the acne stage and the existence of each metabolic- and endocrine-related thyroid precondition, the research found a positive correlation between these diseases and the severity of acne, (*p* < 0.05), regardless of age. Results that were statistically significant showed a positive association between the severity of acne and insulin resistance syndrome, obesity, autoimmune thyroiditis, and autoimmune thyroiditis with hypothyroidism (*p* < 0.05). The metabolic impact of obesity may be connected to the process. Obese patients frequently have higher levels of androgen, insulin, and insulin growth factor, which can stimulate sebaceous cell proliferation and differentiation by expressing adipogenicity genes, resulting in increased sebum output and a change in the severity of acne [37]. Podder et al. observed a 32% greater incidence of MetS in acne subjects, yet when comparing them with the control group, no statistically significant difference was revealed (*p* = 0.06) [38]. In addition, Nagpal et al. noted a higher percentage of acne cases with MetS. In their investigation, 17% of participants in the acne group met MetS parameters, in contrast to 9% in the control group (*p* = 0.09) [39]. In another study, dyslipidemia and insulin resistance were associated with acne in females [40]. In total, 15.4% of the 100 acne-prone women evaluated by Shrestha et al. between 2015 and 2016 had altered lipid profiles [41]. Since androgens are produced from plasma cholesterol and play definite roles in the pathogenesis of acne, lipid changes may be the cause of acne [42]. 

Increased blood glucose levels were linked to acne patients mostly because they cause insulin to be secreted, which reduces the binding protein for IGF-1 and encourages IGF-1 cell proliferation. Increased basal keratinocyte proliferation can lead to acne flare-ups when fasting and postprandial insulin levels are high. Findings suggest that compensatory hyperinsulinemia surpasses IR’s binding capability, allowing insulin to become attached to IGF receptors. The excessive generation of proinflammatory cytokines like C-reactive protein (CRP), interleukin 6 (IL-6), and TNF-α, as well as the malfunction of adipose tissue and compromised adipocyte health, can also result in macrophage infiltration [43]. Additionally, insulin stimulates androgen release, which ultimately results in an increase in sebum production [44]. It is still unclear how thyroid hormones affect the sebaceous glands. Sebocytes in hypothyroid conditions secrete at lower rates [45], and studies have indicated that testosterone co-administration with TSH and thyroxine increases sebum secretion [46]. Alterations in thyroid function parameters related to adult acne were not sufficiently demonstrated [47]. 

Although the precise function of thyroid hormones is yet unknown, it does not appear likely that they are primarily regulated by sebum secretion. Vergou et al. were the first to demonstrate in 2012 that female post-adolescent acne patients suffer from thyroid autoimmunity at much greater rates than healthy subjects [18].

To assess the differences between the control, metabolic preconditioning, endocrine preconditioning-related thyroid disorders, metabolic or endocrine preconditioning, and the metabolic and endocrine preconditioning groups, they were all compared to CP using a chi square test for proportions. The results revealed a significant association (*p* < 0.001), indicating that whenever one or more preconditions are present, the severity of acne increases. 

Growing evidence points to a critical autoinflammatory involvement in the etiology of persistent acne vulgaris. Acne-related autoinflammatory disorders have been shown to share similar pathogeneses, including dysregulated immunity and unusual interleukin-1 signaling, that result in clinically substantial inflammation. Numerous long-term inflammatory skin diseases, such as chronic idiopathic urticaria and acne vulgaris, have been linked to thyroid autoimmunity [48,49]. 

Unfortunately, clinical studies evaluating possible associations between metabolic and endocrine preconditioning-related thyroid disorders, acne stage, and patients’ ages are lacking in the literature. There are many similar pathogenic pathways between acne and the above-mentioned disorders, and comorbidity is a possibility. This comorbidity may take the form of concurrent presence, sequential existence, or mutual causation. 

The risk analyses performed showed an extremely high-risk factor $\left(RR>1,\;95\%\;CI\;RR>1,\;p<0.001\right)$ for developing moderate and severe acne in the presence of the preconditions. When patients have both classes of disease, the chance of developing severe acne is much higher. While the results of the study demonstrated the connections between metabolic and endocrine preconditioning-related thyroid disorders, the acne stages, and the patient’s age, the logistic regression model evaluated the strength of these associations. The outcomes showed a statistically significant association between these variables $\left(p<0.05\right)$. The risk of moderate and severe acne is higher in patients under 30 with metabolic preconditioning and in patients over 30 with endocrine preconditioning. 

This is the first study that, as far as we are aware, assesses the effect of metabolic and endocrine preconditioning-related thyroid disorders on the severity of acne. A correlation between the severity of acne and the presence of various metabolic and endocrine thyroid diseases can be highlighted despite the limitations of this study, including the retrospective investigation and limited sample size for each pathology. The evaluation of clinical and laboratory research related to the risk of metabolic and endocrine diseases in individuals with chronic acne can be highlighted by the findings. The importance of this study also lies in the fact that it emphasizes the need for screening for metabolic and endocrine thyroid disorders in patients with acne vulgaris, such conditions being of the utmost importance to be diagnosed early and treated given their impact on cardiovascular health. The debate regarding the relationship between various metabolic and endocrine preconditions and acne has persisted for a long time, and further research is required to settle it.

## 4. Materials and Methods

### 4.1. Patients and Methods

In this three-year retrospective cohort study, 389 patients diagnosed with acne between January 2020 and December 2022 in a private clinic and private dermatology offices (Pelican Hospital, Oradea, Romania), were assessed to determine the relationship between metabolic and endocrine preconditioning and the severity of the acne. The presence of at least one subcomponent of MetS (i.e., high glucose levels, insulin resistance syndrome, high blood pressure (HBP), dyslipidemia, diabetes, overweight, and obesity) was considered a condition for metabolic preconditioning, and autoimmune thyroiditis, hypothyroidism, or hyperthyroidism (diagnostics established by endocrinologist) were considered endocrine preconditioning.

**Figure 5:**
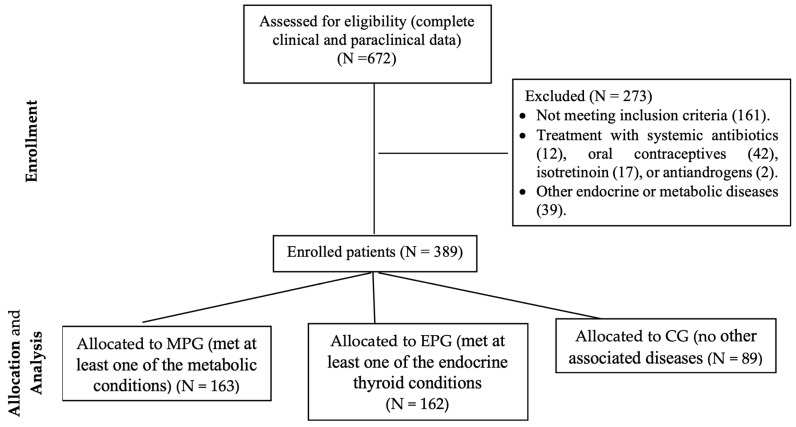
Flow diagram of the study. MPG, metabolic preconditioning group; EPG, endocrine preconditioning group; CG, control group.

In clinic dermatology offices, the medical records of acne patients (N = 672) were examined to determine the patients’ eligibility. Patients who had complete clinical features for acne grading and paraclinical data to establish the presence/absence of preconditions of interest were included in the study: basal glycemia (high glucose level), fasting blood glucose, glycosylated hemoglobin (HbA1c), blood glucose collected at some point of the day (diabetes), HOMA-IR (insulin resistance syndrome), blood pressure (HBP), lipid profile (LDL, HDL, triglycerides) (dyslipidemia), and BMI (weight status) for metabolic preconditioning; and thyroid-stimulating hormone (TSH) (thyroid function), antithyroglobulin antibodies (anti-TG), and/or anti-thyroid peroxidase antibodies (anti-TPO) (autoimmune thyroiditis) for endocrine preconditioning. Of the selected subjects, 389 met the criteria for inclusion in the study, according to the flow chart (Figure 5). 

Patients who met at least one of the criteria described in Table 7 made up the study groups: acne associated with any metabolic disorder was included in the metabolic preconditioning group (denoted MPG, N = 163, 41.9%) or/and acne associated with any endocrine thyroid-related disorder formed the endocrine preconditioning group (EPG, N = 162, 41.65%). Forty-two patients who presented with both metabolic and endocrine disorders simultaneously were included in both groups. Patients diagnosed with acne but without any other associated diseases were included in the control group (CG, N = 89, 22.88%). Patients who were taking systemic antibiotics, oral contraceptives, isotretinoin, or antiandrogens, or who had other endocrine or metabolic diseases, were excluded.

Biochemical parameters were evaluated during the acne diagnosis. After 12 h of fasting, large antecubital veins were venipunctured for collecting blood samples. The samples were handled and evaluated on the same day using identical kits. 

For the patients included in the EPG group, anti-TG, anti-TPO, and TSH were evaluated using electrochemiluminescence immunoassay techniques. The diagnosis of autoimmune thyroiditis was confirmed by determining the level of anti-TG (>115 (UI/mL) and/or anti-TPO (>35 UI/mL), and the results of the thyroid activity were classified as follows:

- Normal thyroid function (TSH = 0.4–4.0 μIU/mL for adults and 0.39–4.0 μUI/mL for women aged between 13 and 20 years);
- Hypofunction (TSH > 4.0 μUI/mL, regardless of sex or age); and
- Hyperfunction (TSH < 0.39 μUI/mL for women aged between 13 and 20 years, or <0.40 μUI/mL for both women/men >20 years old) (Table 7).

Patients with autoimmune thyroiditis who had thyroid hypofunction or hyperfunction were analyzed separately.

For the patients included in the MPG, lipidic and glycemic profiles were considered. Among the analytical determination methods used were the direct colorimetric assessment for HDL and LDL cholesterol, the enzymatic glycerol-3-phosphate oxidase technique for triglycerides, and the oxidase–peroxidase method for cholesterol. Dyslipidemia was defined as an increase in the value of LDL cholesterol (>160 mg/dL), a decrease in the value of HDL cholesterol (<40–50 mg/dL), or an increase in the value of triglycerides (>150 mg/dL). The hexokinase technique was applied to determine the subjects’ basal glucose levels. A normal amount of basal blood glucose was defined as a plasmatic level below 100 mg/dL, whereas a high level of glucose was defined as 100–125 mg/dL. Using a chemiluminescent enzyme immunoassay, serum insulin levels were evaluated. Homeostasis of minimal assessment of insulin resistance (HOMA-IR) was determined based on the basal glucose level and the immunoreactive insulin level using the formula outlined in Table 7. Insulin resistance was characterized by a HOMA-IR score greater than 2. The High-Performance Liquid Chromatography (HPLC) technique was used to determine glycosylated hemoglobin in blood collected on Ethylenediaminetetraacetic acid anticoagulant. A value >6.5% was established for the diagnosis of diabetes. Weight status was determined by calculating BMI [54] and blood pressure was measured according to current guidelines [57]. Overweight and obesity were defined as BMI = 25.00–29.99 kg/m^2^ and >30 kg/m^2^, respectively, and high blood pressure was defined as >130/85 mm Hg (Table 7).

**Figure 6:**
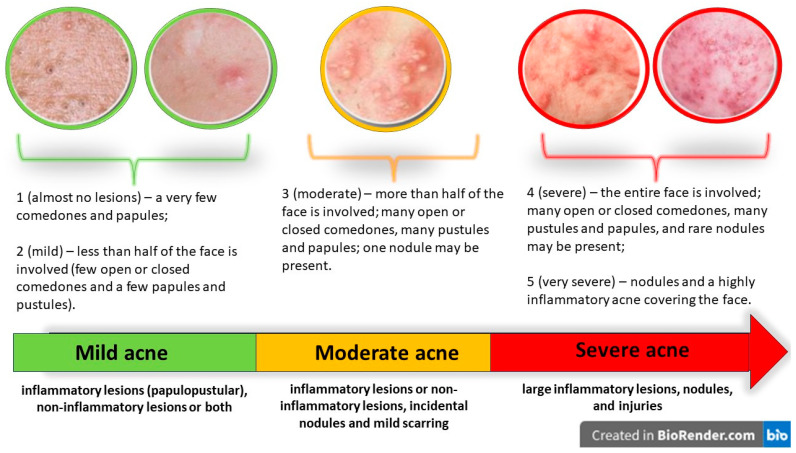
Criteria for acne severity.

The dermatologists used the global acne severity scale (GEA) to assess the severity of the acne according to the criteria mentioned in Figure 6. GEA is a globally validated scale founded on photographs and acne patients, with good agreement in the acne assessments of investigators, and is appropriate for France and Europe [58]. Participants scoring 0, who had no lesions, were removed from consideration.

- Patients scoring 1 or 2 were classified as having “mild acne”, having very few papules and a few sporadic comedones, either closed or open, and simple to identify; the affected area of the face being less than half.
- Patients scoring 3 were classified as having “moderate acne”. This type of acne is characterized by numerous comedones, both closed and open, papules, and pustules, and affects more than half of the face. Or there could be just one nodule, inflammatory and non-inflammatory lesions.
- Patients scoring 4 or 5 had “severe acne”. In these cases, the entire face is affected, with numerous papules and pustules, uncommon nodules, and open or closed comedones. Severe inflammatory injuries throughout the face accompanied by nodules and lesions are also present in these patients.

Depending on the exposure to metabolic or endocrine preconditioning, the severity of acne (as determined by the dermatologists) and the risk of developing more severe acne in the presence of these pathologies were assessed. In the first part of the analysis, when the associations were statistically tested for 3 situations (between a. the age of the patients and the presence of MP and EP related to acne stage/severity for each group; b. the patients’ sex and the presence of MP and EP related to acne stage/severity for each group; and c. the presence of each disease related to acne stage/severity for each group), the patients who simultaneously presented metabolic and endocrine disorders were included both in the MPG and EPG. For evaluating the risk of developing more severe acne in the presence of preconditioning, the differences regarding the frequency of acne according to severity were compared in the groups with associated preconditioning vs. the control group. Moreover, the risk analysis was performed to determine the major risk factors that can favor the development of moderate and severe acne. In this situation, the patients who simultaneously presented metabolic and endocrine disorders were also analyzed separately, consequently creating/resulting the subgroup of patients with both preconditions, denoted MPG and EPG.

### 4.2. Statistical Design

Descriptive statistical analysis, along with frequency tables and representative plots, were used to characterize the database. The Shapiro–Wilk test was applied to see the data distribution for age—the numerical variable, resulting in a *p* value less than 0.05, which implies the use of non-parametrical tests, such as the Mann–Whitney test to see the significance in two separate groups; the Kruskal–Wallis test for more than two different groups; and the chi square test to compare proportions. We also ran a risk analysis to determine the major risk factors that can favor the development of moderate and severe acne. For this, we calculated the Risk Ratio (RR) parameter and estimated the 95% confidence interval (CI). The study ended with a logistic regression analysis, which aims to prove that metabolic and endocrine preconditioning can negatively influence the acne stages. For the entire study, we considered α=0.05 as the significance level. The database was gathered in Microsoft Excel, and the statistical analysis was performed using JASPv17.1 (University of Amsterdam, Department of Psychology and Psychological Methods Unit). The sample size was calculated based on the total number (N = 672) of acne-affected patients who were consulted by a dermatologist during the study period and were diagnosed with acne. A representative sample size of 133 patients was obtained using OpenEpi software, Version 3.01. [59], defining the test power at 80%. 

## 5. Conclusions

Clinicians can observe, diagnose, and track metabolic and endocrine conditions through the lens of the skin. However, in order to establish a correlation between acne and the factors that generate it, a medical visit, consultation, analyses, and a clear diagnosis are needed. The findings of this study highlight a significant association between the severity of acne in young patients and the presence of metabolic and endocrine disorders associated with thyroid preconditioning. Also, our results emphasize the importance of considering the likelihood of metabolic disorders such as insulin resistance syndrome and obesity, as well as thyroid antibodies, in adult patients with acne.

Clinicians are suggested to evaluate adult acne patients for metabolic and endocrine thyroid disorders as part of their diagnostic process. By acknowledging and addressing these underlying variables, healthcare professionals can increase the efficacy of treatment interventions for a wider range of patients.

## Figures and Tables

**Table 1:** The frequency tables pointing out the general characteristics for the whole sample and by groups.

Characteristics	Total (N = 389)		MPC (N = 163)		EPG (N = 162)		CG (N = 89)	
	No.	%	No.	%	No.	%	No.	%
**Gender**								
Female	331	85.1	120	73.62	155	95.68	82	92.13
Male	58	14.9	43	26.38	7	4.32	7	7.87
**Environment**								
Rural	83	21.3	37	22.69	30	18.52	27	30.34
Urban	306	78.7	126	77.31	132	81.48	62	69.66
**Age**								
12–25	93	23.9	69	42.3	5	3.1	19	21.3
>25	296	76.1	94	57.7	157	96.9	70	78.7
Mean age (years)	28.3		25.4		33.9		26.0	
**Acne stage**								
Light	89	22.88	4	2.45	5	3.09	80	89.89
Moderate	155	39.84	92	56.44	58	35.80	5	5.61
Severe	145	37.28	67	41.10	99	61.11	4	4.89
**Concomitant diseases**								
Diabetes	2	0.51	2	1.22	2	1.23	-	-
High glucose	4	1.03	4	2.45	4	2.46	-	-
Insulin resistance syndrome	104	26.73	104	63.80	11	6.79	-	-
Overweight	10	2.57	10	6.13	6	3.70	-	-
Obesity	94	24.16	83	50.92	11	6.79	-	-
Dyslipidemia	13	3.34	7	4.29	6	3.70	-	-
High blood pressure	6	1.54	3	1.84	3	1.85	-	-
Autoimmune thyroiditis	78	20.05	19	11.66	78	20.05	-	-
Hypothyroidism	8	2.06	4	2.45	8	2.06	-	-
Autoimmune thyroiditis + Hypothyroidism	68	17.48	18	11.04	68	41.98	-	-
Hyperthyroidism	-	-	-	-	-	-	-	-
Autoimmune thyroiditis + Hyperthyroidism	9	2.31	1	0.61	9	5.55	-	-

**Table 2:** Descriptive analysis of the age parameter divided by the severity of acne in each sample.

Statistics	Age—Study Group			Age—Control Group			Age—Metabolic Preconditioning Group			Age—Endocrine Preconditioning Group		
	Mild	Moderate	Severe	Mild	Moderate	Severe	Mild	Moderate	Severe	Mild	Moderate	Severe
Mode	24	19	28	25	20	28	24	19	16	29	32	27
Median	25	28	30	25	20	28	28	23	26	27	33.5	34
Mean	26.22	27.42	30.62	26.26	19.6	28.75	28.33	24.28	26.76	27	33.72	34.36
SE	0.55	0.66	0.76	0.62	0.25	0.75	2.6	0.76	1.16	0.95	0.89	0.77
SD	5.27	8.29	9.1	5.53	0.55	1.5	4.51	7.38	9.51	2.12	6.84	7.74
Sh-Wilk	0.89	0.95	0.97	0.89	0.68	0.63	0.99	0.91	0.91	0.91	0.98	0.99
*p* (Sh-ilk)	<0.001	<0.001	<0.001	<0.001	0.006	0.001	0.048	<0.001	<0.001	0.046	0.045	0.036
Range	21	33	37	21	1	3	9	26	37	5	33	37
Minimum	18	14	15	18	19	28	24	14	15	24	14	15
Maximum	39	47	52	39	20	31	33	40	52	29	47	52

SE, standard error of mean; SD, Standard deviation Shapiro–Wilk.

**Table 3:** The association between the patients’ age and the presence of each disease.

Age Split by the Presence/Absence of the Analyzed Variables	Mean Age (Years)	Statistics Mann–Whitney *t* Test	*p* Value
**Metabolic preconditioning**	25.8/30.4	25,461.5	<0.001
High glucose level	31.8/28.3	601.5	0.452
Insulin resistance syndrome	23.3/30.1	22,087.5	<0.001
High blood pressure	44.6/28.2	84.5	0.011
Dyslipidemia	35.4/28.2	790.5	0.02
Diabetes	24.0/28.3	491.5	0.512
Overweight	28.3/28.4	1812.5	0.815
Obesity	24.3/29.4	17,356	<0.001
**Endocrine preconditioning**	33.9/24.3	5841.5	<0.001
Autoimmune thyroiditis	34/26.9	5973	<0.001
Hypothyroidism	25.8/28.4	1755	0.463
Autoimmune thyroiditis + Hypothyroidism	34.5/26.9	5164	<0.001
Hyperthyroidism	-	-	-
Autoimmune thyroiditis + Hyperthyroidism	38.6/28.1	528	0.002

**Table 4:** Patients’ distribution according to acne stage and the existence of each disease.

Group	Acne Stage No./%					
	Mild		Moderate		Severe	
**Control** (89 patients)	80	89.89	5	5.62	4	4.49
**Metabolic preconditioning** (163 patients)	4	2.45	92	56.44	67	41.1
Glucose	-	-	-	-	4	5.97
Insulin resistance syndrome	-	-	63	68.49	41	61.19
High blood pressure	-	-	-	-	3	4.49
Dyslipidemia	1	25	2	2.17	5	7.46
Diabetes	-	-	-	-	2	2.99
Overweight	3	75	4	4.35	4	5.97
Obesity	-	-	66	71.74	17	25.37
**Endocrine preconditioning** (162 patients)	5	3.09	58	35.8	99	61.11
Autoimmune thyroiditis	1	20	26	44.83	51	51.52
Hypothyroidism	3	60	5	8.62	-	-
Autoimmune thyroiditis + Hypothyroidism	-	-	24	41.38	44	44.44
Hyperthyroidism	-	-	-	-	-	-
Autoimmune thyroiditis + Hyperthyroidism	1	20	4	6.89	4	4.04

**Table 5:** The association between the severity of acne and the presence of each disease (when there are less than two subjects in a group, the chi square test cannot be applied).

Acne Stage Split by the Presence/Absence of the Analyzed Variables	$\mathrm{Statistics}\;\mathrm{of}\;\chi^{2}$ Test	*p* Value	$\mathrm{Statistics}\;\mathrm{of}\;\chi^{2}$ Test 12–25 Years	*p* Value	$\mathrm{Statistics}\;\mathrm{of}\;\chi^{2}$ Test >25 Years	*p* Value
**Metabolic preconditioning**	84.897	<0.001	51.859	<0.001	78.360	<0.001
Glucose	7.109	0.029	-	-	7.109	0.029
Insulin resistance syndrome	47.161	<0.001	28.331	<0.001	44.770	<0.001
High blood pressure	5.318	0.07	-	-	5.318	0.07
Dyslipidemia	4.338	0.114	-	-	4.356	0.113
Diabetes	3.536	0.171	2.347	0.309	3.536	0.171
Overweight	0.48	0.959	-	-	0.476	0.788
Obesity	70.958	<0.001	25.818	<0.001	73.064	<0.001
**Endocrine preconditioning**	97.110	<0.001	2.607	0.272	98.268	<0.001
Autoimmune thyroiditis	44.607	<0.001	2.415	0.299	40.728	<0.001
Hypothyroidism	4.645	0.098	0.971	0.615	4.617	0.099
Autoimmune thyroiditis + Hypothyroidism	38.377	<0.001	-	-	34.508	<0.001
Hyperthyroidism	-	-	-	-	-	-
Autoimmune thyroiditis + Hyperthyroidism	0.845	0.655	-	-	0.869	0.647

**Table 6:** Risk analysis run on the patients with acne (2 × 2 tables).

The Contingency Tables	Stage of Acne		Statistics
**Samples**	Moderate	Mild	$\begin{array}{c} RR=16.29>1\\ 95\%\;CI\;RR\in \left(6.95;38.17\right)\\ p<0.001 \end{array}$
Metabolic preconditioning	92	4	
Control	5	80	
Metabolic preconditioning	Severe	Mild	$\begin{array}{c} RR=19.82>1\\ 95\%\;CI\;RR\in \left(7.6;51.66\right)\\ p<0.001 \end{array}$
	67	4	
Control	4	80	
Endocrine preconditioning	Moderate	Mild	$\begin{array}{c} RR=15.65>1\\ 95\%\;CI\;RR\in \left(6.66;36.74\right)\\ p<0.001 \end{array}$
	58	5	
Control	5	80	
Endocrine preconditioning	Severe	Mild	$\begin{array}{c} RR=19.99>1\\ 95\%\;CI\;RR\in \left(7.67;52.07\right)\\ p<0.001 \end{array}$
	99	5	
Control	4	80	
Metabolic and endocrine preconditioning	Moderate	Mild	$\begin{array}{c} RR=15.45>1\\ 95\%\;CI\;RR\in \left(6.47;36.91\right)\\ p<0.001 \end{array}$
	10	1	
Control	5	80	
Metabolic and endocrine preconditioning	Severe	Mild	$\begin{array}{c} RR=20.34>1\\ 95\%\;CI\;RR\in \left(7.8;53.05\right)\\ p<0.001 \end{array}$
	31	1	
Control	4	80	

**Table 7:** Criteria for inclusion in the study groups.

Criteria by Groups	Reference Ranges	Ref.
**Metabolic preconditioning group (N = 163)**		
High glucose level: Basal glycemia	100–125 mg/dL	[50]
Insulin resistance syndrome: HOMA-IR = (insulin (µU/mL) × glucose level (mg/dL))/405.	>2	[51]
High blood pressure	>130/85 mm Hg	[50]
Dyslipidemia • Increase in the value of LDL cholesterol • Decrease in the value of HDL cholesterol • Increasing the value of triglycerides	>160 mg/dL <40–50 mg/dL >150 mg/dL	[52] [50] [50]
Diabetes • Fasting blood glucose • Glycosylated hemoglobin (HbA1c) • Blood glucose collected at some point of the day	>/=126 mg/dL >/=6.5% >/=200 mg/dL	[53]
Overweight: Body mass index	25.00 to 29.99 kg/m^2^	[54]
Obesity: Body mass index	>30 kg/m^2^	[54]
**Endocrine preconditioning group (N = 162)**		
Autoimmune thyroiditis * • Antithyroglobulin antibodies (anti-TG) • Anti-thyroid peroxidase antibodies (anti-TPO)	anti-TG > 115 (UI/mL) and/or anti-TPO > 35 (UI/mL)	[55]
Hyperthyroidism *: thyroid-stimulating hormone (TSH)	<0.39 (μUI/mL), women, aged between 13–20 years <0.40 (μUI/mL), for both women/men, >20 years	[56]
Hypothyroidism *: thyroid-stimulating hormone (TSH)	>4.0 (μUI/mL), regardless sex or age	[56]

* The reference range, established by the laboratory protocol.

## Data Availability

Data are contained within the article.
